# Risk of Adverse Pregnancy Outcomes among Women Practicing Poor Sanitation in Rural India: A Population-Based Prospective Cohort Study

**DOI:** 10.1371/journal.pmed.1001851

**Published:** 2015-07-07

**Authors:** Bijaya K. Padhi, Kelly K. Baker, Ambarish Dutta, Oliver Cumming, Matthew C. Freeman, Radhanatha Satpathy, Bhabani S. Das, Pinaki Panigrahi

**Affiliations:** 1 Asian Institute of Public Health, Bhubaneswar, India; 2 College of Public Health, University of Iowa, Iowa City, Iowa, United States of America; 3 Faculty of Infectious and Tropical Diseases, London School of Hygiene & Tropical Medicine, London, United Kingdom; 4 Rollins School of Public Health, Emory University, Atlanta, Georgia, United States of America; 5 College of Public Health, University of Nebraska Medical Center, Omaha, Nebraska, United States of America; Kings College London, United Kingdom

## Abstract

**Background:**

The importance of maternal sanitation behaviour during pregnancy for birth outcomes remains unclear. Poor sanitation practices can promote infection and induce stress during pregnancy and may contribute to adverse pregnancy outcomes (APOs). We aimed to assess whether poor sanitation practices were associated with increased risk of APOs such as preterm birth and low birth weight in a population-based study in rural India.

**Methods and Findings:**

A prospective cohort of pregnant women (*n* = 670) in their first trimester of pregnancy was enrolled and followed until birth. Socio-demographic, clinical, and anthropometric factors, along with access to toilets and sanitation practices, were recorded at enrolment (12th week of gestation). A trained community health volunteer conducted home visits to ensure retention in the study and learn about study outcomes during the course of pregnancy. Unadjusted odds ratios (ORs) and adjusted odds ratios (AORs) and 95% confidence intervals for APOs were estimated by logistic regression models. Of the 667 women who were retained at the end of the study, 58.2% practiced open defecation and 25.7% experienced APOs, including 130 (19.4%) preterm births, 95 (14.2%) births with low birth weight, 11 (1.7%) spontaneous abortions, and six (0.9%) stillbirths. Unadjusted ORs for APOs (OR: 2.53; 95% CI: 1.72–3.71), preterm birth (OR: 2.36; 95% CI: 1.54–3.62), and low birth weight (OR: 2.00; 95% CI: 1.24–3.23) were found to be significantly associated with open defecation practices. After adjustment for potential confounders such as maternal socio-demographic and clinical factors, open defecation was still significantly associated with increased odds of APOs (AOR: 2.38; 95% CI: 1.49–3.80) and preterm birth (AOR: 2.22; 95% CI: 1.29–3.79) but not low birth weight (AOR: 1.61; 95% CI: 0.94–2.73). The association between APOs and open defecation was independent of poverty and caste. Even though we accounted for several key confounding factors in our estimates, the possibility of residual confounding should not be ruled out. We did not identify specific exposure pathways that led to the outcomes.

**Conclusions:**

This study provides the first evidence, to our knowledge, that poor sanitation is associated with a higher risk of APOs. Additional studies are required to elucidate the socio-behavioural and/or biological basis of this association so that appropriate targeted interventions might be designed to support improved birth outcomes in vulnerable populations. While it is intuitive to expect that caste and poverty are associated with poor sanitation practice driving APOs, and we cannot rule out additional confounders, our results demonstrate that the association of poor sanitation practices (open defecation) with these outcomes is independent of poverty. Our results support the need to assess the mechanisms, both biological and behavioural, by which limited access to improved sanitation leads to APOs.

## Abstract 2

Pinaki Panigrahi and colleagues examine the association between adverse pregnancy outcomes and sanitation practices in pregnant women in rural India.

## Editors' Summary

**Background:**

Pregnancy is usually a happy time for women and their families. But, for some women, pregnancy ends unhappily. Some women lose their baby during early pregnancy (spontaneous abortion or miscarriage) or during late pregnancy (stillbirth). Others have their baby earlier than expected (preterm birth) or have a baby with low birth weight, two outcomes that adversely affect the baby’s survival and long-term health. The burden of adverse pregnancy outcomes (low birth weight, preterm birth, stillbirth, and spontaneous abortion) is substantial across the world but is particularly high in resource-limited settings. More than 60% of all preterm births take place in Asia and sub-Saharan Africa, and in India alone nearly 13 million babies (47% of all births) had a low birth weight in 2010. Many risk factors for adverse pregnancy outcomes have been identified, including infection, diabetes, poor antenatal care, and other socio-economic factors, but a clear causal mechanism for adverse pregnancy outcomes has not been established.

**Why Was This Study Done?:**

One potential risk factor for adverse pregnancy outcomes, particularly in resource-limited settings, is poor sanitation—the inadequate provision of facilities and services for the safe disposal of human urine and feces. The WHO/UNICEF Joint Monitoring Programme for Water Supply and Sanitation estimates that, globally, 1.1 billion people defecate in the open, a practice that can expose individuals to contact with human feces containing infectious organisms and that can contaminate food and water. Poor sanitation might contribute to adverse pregnancy outcomes by promoting infection or by causing stress during pregnancy. Women might, for example, limit their intake of food and water to avoid having to use inadequate toilet facilities, thereby adversely affecting the health of their unborn child. Here, the researchers assess whether poor sanitation practices are associated with an increased risk of adverse pregnancy outcomes by undertaking a population-based prospective study in two rural areas of Odisha state, India. Odisha has a high infant death rate (57 deaths per 1,000 live births), only 18.2% of households have access to an improved latrine (a facility such as a flush toilet that hygienically prevents human contact with human excreta), and 75% of households practice open defecation.

**What Did the Researchers Do and Find?:**

For their study, the researchers enrolled 670 women during the first trimester of their pregnancy. They recorded socio-demographic data (for example, age, level of education, and household assets), clinical data, weight and height, and toilet access and sanitation practices for each woman at enrollment and followed them through pregnancy until birth. Nearly two-thirds of the women practiced open defecation, and a quarter experienced an adverse pregnancy outcome, most commonly a preterm birth and/or having a baby with low birth weight. After adjustment for potential confounding factors (factors that might affect outcomes, such as socio-demographic characteristics), open defecation was significantly associated with adverse pregnancy outcomes (all four adverse outcomes considered together) and with preterm birth, but not with low birth weight (a significant association is one that is unlikely to have happened by chance). Specifically, the adjusted odds ratios (an indicator of the strength of association between an exposure and an outcome; an odds ratio of more than one indicates that an exposure increases the risk of an outcome) of adverse pregnancy outcomes and preterm birth among women practicing open defecation compared with women with access to a latrine were 2.38 and 2.22, respectively. Notably, these associations were independent of poverty, caste, and religion.

**What Do These Findings Mean?:**

These findings indicate that, among women in Odisha, defecation in the open (poor sanitation) during pregnancy is associated with a higher risk of any adverse pregnancy outcome and of preterm birth than the use of a latrine. Counterintuitively, these findings also suggest that the association between open defecation and adverse pregnancy outcomes is not explained by poverty. Although the researchers adjusted for numerous confounding factors in their analysis, the women who defecated in the open may have shared some other unknown characteristic (residual confounding) that was actually responsible for their increased risk of an adverse pregnancy outcome. Further studies are now needed to determine the socio-behavioral and/or biological basis of the association between poor sanitation and adverse pregnancy outcomes. Appropriate public health interventions can then be designed to reduce the burden of adverse pregnancy outcomes among women living in settings where there is limited access to adequate sanitation.

**Additional Information:**

This list of resources contains links that can be accessed when viewing the PDF on a device or via the online version of the article at http://dx.doi.org/10.1371/journal.pmed.1001828.

- The March of Dimes, a non-profit organization for pregnancy and baby health, provides information on pregnancy loss, preterm birth, and low birth weight
- Tommy’s, a UK non-profit organization that funds research into stillbirth, premature birth, and miscarriage, also provides information about adverse pregnancy outcomes
- The World Health Organization (WHO) provides information on water, sanitation, and health (in several languages)
- The WHO/UNICEF Joint Monitoring Programme for Water Supply and Sanitation monitors progress toward improved global sanitation; its 2014 report on progress in water sanitation is available (in several languages)
- The children’s charity UNICEF, which protects the rights of children and young people around the world, provides information on water, sanitation, and health (in several languages)
- The Water Supply and Sanitation Collaborative Council and the non-governmental organization Practical Action provide information on approaches and technologies for improving sanitation
- A *PLOS Medicine* Collection on water and sanitation and a Policy Forum by Velleman et al. on improving water, sanitation, and hygiene for maternal and newborn health are available

## Introduction

The burden of adverse pregnancy outcomes (APOs), which includes both preterm births and low birth weights [1,2], is substantial in both developed and developing countries [1–3]. More than 60% of preterm births take place in south Asia and sub-Saharan Africa [3]. A recent study estimated that 12.8 million babies were born small for gestational age in India alone in the year 2010, a prevalence of 47% of all births [1]. Preterm birth and low birth weight are critical determinants of child survival, disabilities, stunting, and long-term adverse consequences for the onset of non-communicable diseases in the life course and demand appropriate public health interventions [1,4]. Despite India’s impressive economic growth in the last two decades, access to improved sanitation services in rural and vulnerable communities is extremely limited.

The World Health Organization (WHO) defines a birth weight of <2,500 g as low birth weight and a delivery before 37 completed weeks of gestation as preterm birth [5]. We adopted the WHO guidelines that define an APO as an event of low birth weight, preterm birth, stillbirth, or abortion. APO is a complex, multifactorial, physiological outcome in women, and despite decades of research, a clear causal mechanism for APOs has not been established. Studies have reported numerous risk factors for APOs such as malaria [6], infection [7–12], anaemia [13–16], obesity [17], hypertension [18], hyperglycaemia [19], diabetes [20], periodontal disease [21], endometriosis [22], history of abortion [23], antenatal complications [24], antenatal care (ANC) [24], environmental pollution [25–29], violence [30], and other socio-economic disparities [31–33]. In many low- and middle-income countries, access to improved sanitation facilities is limited, but the link between sanitation and APOs has not been explored.

The WHO/UNICEF Joint Monitoring Programme for Water Supply and Sanitation (JMP) defines an improved sanitation facility as a facility that hygienically separates human excreta from human contact, such as a flush toilet, piped sewer system, septic tank, flush/pour flush to pit latrine, ventilated improved pit latrine, pit latrine with slab, or composting toilet [34]. Similarly, the JMP defines unimproved sanitation facilities as flush/pour flush to elsewhere, pit latrine without slab, bucket, hanging toilet or hanging latrine, no facilities, or bush or field. Globally, 1.1 billion people still practices open defecation, of which 638 million are in India [34]. Poor sanitation, alongside unsafe drinking water and hygiene, are responsible for a considerable proportion of the global burden of disease [35,36]. Water, sanitation, and hygiene (WASH) interventions have been linked to improvements in a number of important health outcomes, including diarrhoeal diseases [37], helminth infections [38,39], and childhood stunting [40,41]. Recent studies in India have failed to show that programmes to improve sanitation in India lead to health gains in children aged under 5 y, although critical in these findings were the low levels of improved sanitation use among the population [42,43]. Although little work has been done to evaluate the effects of WASH interventions on APOs, a recent review identified over 60 biological and social mechanisms linking poor WASH practices to various maternal and reproductive health outcomes [44].

At least some of these identified plausible mechanisms linking open defecation to APOs are supported by existing evidence. For example, there is good evidence that poor sanitation can promote hookworm infestation [39], which is a risk factor for maternal anaemia [10,45,46], which, in turn, is directly linked to APOs [16,47]. In India, the prevalence of anaemia and chronic energy deficiency (measured as low body mass index [BMI]) in women aged 15–49 y is as high as 55.3% and 35.6%, respectively [48,49].

Exposure to unsafe water, unimproved sanitation, and poor waste management during pregnancy may increase the risk of infection, causing downstream effects such as low birth weight and preterm delivery. A recent systematic review [50] and a conceptual framework [44] concluded that a lack of improved sanitation facilities appears to be associated with maternal mortality, and highlighted the paucity of primary studies assessing the impact of water and sanitation practices on pregnancy outcomes [44,50].

To the best of our knowledge, this is the first rigorous attempt to quantify the risk of APOs with access to improved sanitation and practice using a population-based cohort, with specific aims to quantify the prevalence of open defecation among pregnant women and its association with APOs.

## Methods

### Ethical Approval

Ethical approval for the study was obtained from the Ethical Review Committee of the Asian Institute of Public Health(Bhubaneswar, India), and the Institutional Review Board at Emory University (Atlanta, Georgia). Written informed consent to participate in the study was obtained from each study participant at the time of recruitment. Participants were informed about the purpose of the study, and that they were free to withdraw from the study at any point. The survey team also received cultural competency and confidentiality training from a qualified trainer.

### Study Settings and Participants

The state of Odisha, home to 41.9 million people, has one of the highest infant mortality rates (53 per 1,000 live births) and maternal mortality rates (235 per 100,000 women of reproductive age) in India [51]. Odisha faces a number of serious challenges: frequent natural disasters, high levels of unemployment, and over 40% of the population living below the poverty line [51]. In 2011, only 18.2% of households had access to an improved latrine, and more than 75% of households practiced open defecation [52].

Since improved sanitation access and uses are associated with class, caste, and geography [53,54], we attempted to include a diverse and representative sample by including two geographically predominant and distinct areas of Odisha state (S1 Fig). Lathikata and Kuarmunda, the administrative revenue blocks of Sundargarh, a northwest inland tribal district, and Balianta and Balipatana, the revenue blocks of Khurda, a typical rural district located on the east coast of the country, were chosen for this study. Villages in the inland tribal populations (Sundargarh study setting) are spread over hilly and mining areas where communities follow their traditional lifestyle with minimal outside interaction. In contrast, inhabitants of the coastal rural population (Khurda study setting) depend on irrigated agriculture, farming, and small-scale business. Some individuals work in government offices and other small service industries. Individuals in the Khurda study setting are relatively more affluent and live in densely populated villages that are close to each other.

For the current study, we utilised an existing population-based surveillance cohort with a combined population of 360,000, with approximately 60,000 married women of reproductive age (13–49 y). This cohort was established for the recently completed Aetiology of Neonatal Infection in South Asia (ANISA) study [55], where all pregnancies were being tracked and recorded using the GPS coordinates of the mothers’ homes. A small subset of pregnant women was randomly chosen from this existing cohort for the current study. We trained our community health volunteers (CHVs), women from the same villages as the participants, to recruit eligible pregnant women in our study. Pregnant women (10–12 wk of gestation) who were residents of the locality, who were between 18 and 48 y of age, and who provided informed consent were eligible to participate in the study. We followed a three-tiered monitoring and reporting system. Each study setting (Sundargarh and Khurda) had one CHV responsible for following 4–5 pregnancies, supervised by an area coordinator (one per ten CHVs), who reported to a programme manager. Our CHVs worked very closely with government personnel, including Anganwadi workers and Accredited Social Health Activists, of the study settings.

### Study Design

**Fig 1:**
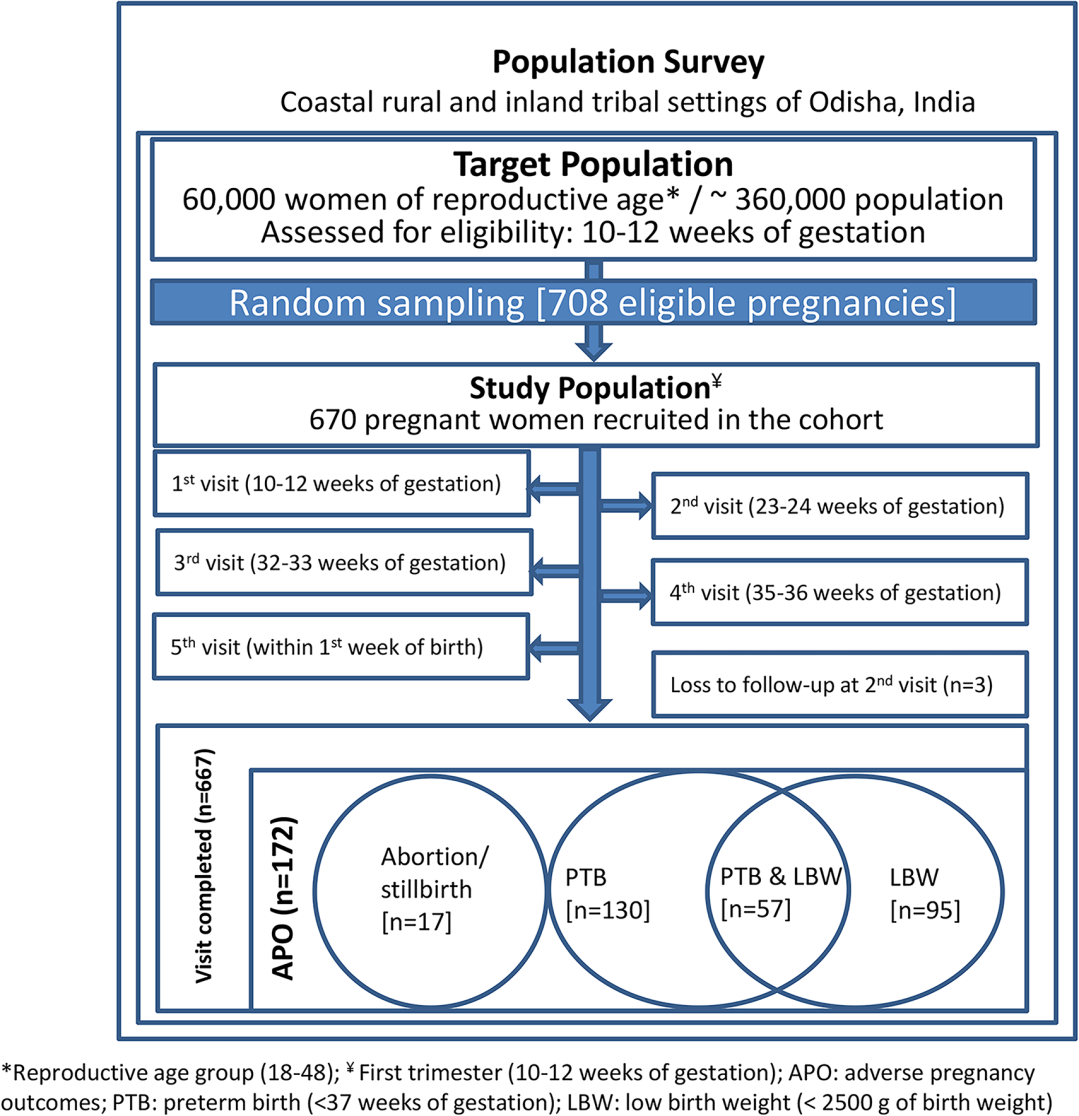
Study design and sampling scheme.

We conducted a population-based prospective cohort study. Assuming a 20.0% prevalence of APOs [24,56–58], we calculated that the sample size needed to be 582 to detect a relative risk (the difference in incidence of APOs between women with and without improved sanitation) of 1.5 with 95% confidence at 80% power. All pregnant women satisfying the eligibility criteria in the study population were recruited into the study. Estimating an anticipated 15% dropout rate, our final target sample size was 670. There were 708 eligible pregnant women in the two study settings (coastal and inland). A random number generator selected the 670 geocoded households/women who were enrolled through a household visit by the CHV. A schematic diagram of the study design is shown in Fig 1. Baseline assessment at recruitment and three home visits (one in the second trimester and two in the third trimester) were designed to ensure retention and learn about study outcomes during the course of pregnancy, followed by a home visit at birth to document pregnancy outcomes. Sanitation exposures measured at baseline were used to estimate the risks of pregnancy outcomes.

### Exposure Measures

The survey instruments (questionnaires and observation checklists) were developed in three stages. First, a preliminary survey questionnaire was developed after literature review and inputs from key stakeholders. Second, a draft sanitation exposure assessment questionnaire was developed through focus group discussions with selected pregnant women and key informants in the community. Third, a pre-test was conducted using the preliminary questionnaire in nearby non-study villages to prevent contamination. After triangulation, the main survey administered by the trained CHVs addressed specific questions on sanitation and hygiene practices and conditions during recruitment. Visual observations of defecation sites were performed to confirm the interview response. Where a latrine was used, observational checklists were also used to inspect for the presence of a functional water source or water storage container at the latrine, type of latrine, visible faecal contamination on latrine floors, and presence of a hand-washing station with soap, detergent, or ash at or near the toilet. Other information, such as hand-washing practice after defecation and source of bathing water, was also collected. We defined “poor sanitation” according the JMP criteria of unimproved sanitation facilities and latrine use behaviour [34].

### Outcome Measures

We used the WHO definitions for all outcome measures [5]. The primary outcome of interest was incidence of an APO, defined as an event of preterm birth, low birth weight, spontaneous abortion, or stillbirth [5]. Infant demographics such as gestational age and birth weight were abstracted from medical charts at delivery by the CHV. Gestational age was ascertained by the dating method from the last menstrual period. Our female field personnel were extensively trained with the lunar calendar (used by the women in Odisha state) and how to record the first day of the last menstrual period. In our study settings, all of the deliveries were conducted at health centres by a qualified health care service provider.

### Potential Covariates and Confounders

Household socio-demographic information, including maternal age at enrolment, education, religion, caste, and previous pregnancy history, was collected by CHVs using a structured instrument in face-to-face interviews. Since most of the pregnant women had limited knowledge on their family’s monthly income, we derived an indirect measure of household economic information from household characteristics and asset data [59] obtained at enrolment. Household characteristics included type of house, household electrification, drinking water source, cooking fuel, light source for household, number of rooms used for sleeping, ownership of agricultural land, whether agricultural land was irrigated or not, ownership of business establishments, and household assets included radios, televisions, fans, mobile telephones, refrigerators, bicycles, motorcycles/scooters, and cars. During the enrolment visit, the height and weight of the participants were measured using standard protocols and digital weighing machines calibrated in the field study office every morning. Relevant data on haemoglobin (Hb) was obtained from the Mother & Child Tracking System card (issued by the Ministry of Health and Family Welfare to pregnant women) of the participants. For women for whom this information was unavailable, a study supervisor conducted the haemoglobin estimation from finger prick blood samples using a portable haemoglobin analyzer (HemoCue Hb 301). Pregnant women’s ANC coverage at birth was also recorded.

### Data Collection and Confidentiality

The survey instruments, including the informed consent document, were translated into the local language (Odiya) and administered by the trained female CHVs. A unique study ID was assigned to each participant and used subsequently on all study forms. Surveys were administered inside the home of the participants, and all study forms, including the informed consent document, were transported by supervisors to a field office and then on to the study hospitals for storage in secure, locked metal cabinets. All personal identifiers were removed from the dataset and were kept along with the informed consent documents securely under the custody of the principal investigator. Only trained personnel had access to the rest of the survey documents for data entry and management.

### Data Quality Assurance

The survey instruments were piloted in villages from similar settings that were not included in the study. The quality of the data collected by the CHVs was ensured through direct supervision by respective field supervisors and subsequently by the programme manager. Supervisory visits and standardisation exercise sessions were organised routinely to ensure the quality of the data collected. Every reported outcome of interest was confirmed by a repeat visit to the household by supervisory staff. CHVs submitted data forms to their supervisor, who checked the forms for completeness and consistency. Double entry was done for all study forms into a custom-designed database management interface using the EpiInfo platform. The quality of the primary outcome measures (gestational age and birth weight) was ensured through a quality indicator of high gestational age at birth (i.e., ≥44 wk); all birth weights were abstracted from medical charts at delivery. The quality of key exposure data (latrine access and use) was crosschecked with observational data supporting use of latrine or open defecation. For analysis and reporting purposes, all of the dataset passed the above quality assurance measures.

### Statistical Analysis

We conducted cross-tabulation to explore frequencies and bivariate associations between key independent variables and the outcomes (APO, preterm birth, and low birth weight). The main independent variable (sanitation) was categorised into access to a latrine (private or neighbour’s) versus open defecation.

We used principal component analysis with varimax rotation for computing a wealth index [59] from the household characteristics and asset data. Based on the distribution of the wealth index, the households were then divided into four groups (quartiles) of socio-economic status: low, lower medium, upper medium, and high (S1 Table).

We estimated unadjusted odds ratios (ORs) and adjusted odds ratios (AORs) of the relationship between improved sanitation access and APOs using logistic regression models. Our predefined analysis using a generalized linear model was changed to logistic regression in response to reviewer requests and because of the types of data included in the model (S1 Text).

We included a priori covariates—such as materials used to wash hands after defecation, source of bathing water, place of residence, maternal age, maternal parity, BMI, maternal haemoglobin, ANC, poverty, educational level, caste, and religion—for APOs that were thought to be important confounding factors on theoretical grounds [60]. The covariate poverty ascertained from below poverty line status was changed to the wealth index at the peer-review stage. Where applicable, we incorporated a categorical/continuous parameterization into the multivariate model to better control for confounders. Data were analysed using STATA (version 13).

## Results

Of the 670 women recruited, 667 completed the study, of which 172 (28.2%) experienced APOs (Fig 1). Among these women, 130 (19.4%) had preterm births, 95 (14.2%) gave birth to babies with low birth weight, 11 (1.6%) had spontaneous abortions, and six (0.9%) had stillbirths. Detailed socio-demographic, anthropometric, and clinical characteristics of study participants stratified by APO are shown in Table 1. In our study population, about 85% of the women were 20–29 y-old, 72% had normal BMI (range 18.5–24.9 kg/m^2^), 36.1% did not have anaemia (Hb ≥ 110 g/l), 35.4% were primiparous, and 15.1% had no formal education (Table 1).

**Table 1:** Pregnant women’s selected socio-demographic, anthropometrics, and clinical characteristics stratified by adverse pregnancy outcome (*n* = 667).

Characteristic	All Participants	Low Birth Weight (*n* = 95)	Preterm Birth (*n* = 130)	APO (*n* = 172)
**Maternal age**				
<20 y	25 (3.75)	4 (4.21)	4 (3.08)	10 (5.81)
20–24 y	326 (48.88)	49 (51.58)	68 (52.31)	86 (50.00)
25–29 y	234 (35.08)	21 (22.11)	37 (28.46)	49 (28.49)
30–34 y	55 (8.25)	5 (5.26)	10 (7.69)	11 (6.40)
≥35 y	27 (4.05)	16 (16.84)	11 (8.46)	16 (9.30)
**Maternal BMI**				
Underweight (<18.5 kg/m^2^)	141 (21.14)	30 (31.58)	36 (27.69)	49(28.49)
Overweight (≥25.0 kg/m^2^)	43 (6.45)	2 (2.11)	6 (4.62)	6 (3.49)
Normal weight (18.5–24.9 kg/m^2^)	483 (72.41)	63 (66.32)	88 (67.69)	117 (68.02)
**Maternal haemoglobin**				
Anaemic (Hb < 110 g/l)	426 (63.87)	75 (78.95)	101 (77.69)	135 (78.49)
Not anaemic (Hb ≥ 110 g/l)	241 (36.13)	20 (21.05)	29 (22.31)	37 (21.51)
**Maternal parity**				
0	236 (35.38)	39 (41.05)	49 (37.69)	69 (40.12)
1	61 (09.15)	9 (9.47)	15 (11.54)	17 (9.88)
2	99 (14.84)	16 (16.84)	19 (14.62)	27 (15.70)
≥3	271 (40.63)	31 (32.63)	47 (36.15)	59 (34.30)
**Maternal education**				
No education	101 (15.14)	21 (22.11)	33 (25.38)	43 (25.00)
Incomplete primary	131 (19.64)	37 (38.95)	36 (27.69)	52 (30.23)
Complete primary	161 (24.14)	17 (17.89)	27 (20.77)	35 (20.35)
Incomplete secondary	119 (17.84)	7 (7.37)	12 (9.23)	14 (8.14)
Complete secondary	101 (15.14)	9 (9.47)	14 (10.77)	19 (11.05)
Higher	54 (8.10)	4 (4.21)	8 (6.15)	9 (5.23)
**Wealth index quartile** *				
Low	169 (25.34)	14 (14.74)	27 (20.77)	32 (18.60)
Lower medium	165 (24.74)	23 (24.21)	33 (25.38)	46 (26.74)
Upper medium	168 (25.19)	43 (45.26)	48 (36.92)	66 (38.37)
High	165 (24.74)	15 (15.79)	22 (16.92)	28 (16.28)
**Place of residence**	** **	** **	** **	** **
Coastal (rural)	203 (30.43)	23 (24.21)	24 (18.86)	35 (20.35)
Inland (tribal)	464 (69.57)	72 (75.79)	106 (81.54)	137 (79.65)
**ANC visits completed at birth**	** **	** **	** **	** **
One	28 (4.20)		11 (8.54)	11 (6.40)
Two	141 (21.14)	36 (37.89)	89 68.46)	96 (55.81)
Three	498 (74.66)	59 (62.11)	30 (23.08)	65 (37.79)
**Religion**				
Hindu	226 (33.88)	25 (26.32)	30 (23.08)	41 (23.84)
Muslim	69 (10.34)	9 (9.47)	13 (10.00)	17 (9.88)
Christian	372 (55.77)	61 (64.21)	87 (66.92)	114 (66.28)
**Social caste**				
General	113 (16.94)	9 (9.47)	15 (11.54)	20 (11.63)
Other backward class	71 (10.64)	8 (8.42)	10 (7.69)	14 (8.14)
Schedule caste	102 (15.29)	17 (17.89)	20 (15.38)	27 (15.70)
Schedule tribe	381 (57.12)	61 (64.21)	85 (65.38)	111 (64.53)

Data are given as *n* (percent).

*Principal component analysis with varimax rotation was used for computing the wealth index. Based on the distribution of the wealth index, the households were then divided into four groups (quartiles).

Table 2 provides the prevalence of sanitation access and practices with unadjusted ORs for APOs. Of the 667 women, 388 (58.2%) had no access to a latrine and reported open defecation at recruitment. About half (45.8%) of the pregnant women living in a household with latrine access used the latrine on a regular basis, and 32% reported rare use of the facility. The majority (72.4%) of the latrine facilities were simple pit latrines. About 60% of the latrines had a water source in or at the latrine, and 21.5% of the latrines had visible faecal contamination on the latrine floor (an indication of poor sanitation practice). About half of the households had a hand-washing station with soap at or near the latrine. In our population, 58% of pregnant women did not wash their hands with soap or detergent after defecation. Only 14.7% of participants in our study used piped water for bathing or body washing.

**Table 2:** Pregnant women’s sanitation access and use with unadjusted odds ratios for adverse pregnancy outcomes.

Sanitation Characteristic	*n* (Percent)	Low Birth Weight (*n* = 95)		Preterm Birth (*n* = 130)		APO (*n* = 172)	
		OR (95% CI)	*p*-Value	OR (95% CI)	*p*-Value	OR (95% CI)	*p*-Value
**Access to latrine (*n* = 667)**							
Latrine in house/neighbour’s house	279 (41.83)	Ref.		Ref.		Ref.	
No latrine—go to open field/bush	388 (58.17)	2.00 (1.24–3.23)	0.004	2.36 (1.54–3.62)	<0.001	2.53 (1.72–3.71)	<0.001
**Latrine type (observed) (*n* = 279)**							
Ventilated improved pit latrine	46 (16.49)	Ref.		Ref.		Ref.	
Flush/pour flush to septic tank	31 (11.11)	1.53 (0.28–8.15)	0.412	0.98 (0.15–6.28)	0.990	0.87 (0.19–3.97)	0.867
Simple pit latrine/composting/dry latrine	202 (72.40)	1.69 (0.48–5.93)	0.615	2.40 (0.69–8.25)	0.164	1.83 (0.68–4.97)	0.230
**Self-reported latrine use (*n* = 279)**							
Often/daily	128 (45.88)	Ref.		Ref.		Ref.	
Few times a week	62 (22.22)	1.33 (0.41–4.27)	0.024	2.20 (0.73–6.57)	0.158	2.04 (0.81–5.11)	0.126
Rarely/as needed/only if someone is sick	89 (31.90)	2.87 (1.15–7.19)	0.622	5.01 (2.01–12.44)	0.001	3.92 (1.80–8.52)	0.001
**Water available at latrine (observed) (*n* = 279)**							
Yes	172 (61.65)	Ref.		Ref.		Ref.	
No	107 (38.35)	3.16 (1.38–7.21)	0.006	4.68 (2.13–10.25)	<0.001	4.07 (2.07–8.02)	<0.001
**Visible faecal contamination at latrine (observed) (*n* = 279)**							
No	219 (78.49)	Ref.		Ref.		Ref.	
Yes	60 (21.51)	3.16 (1.38–7.21)	0.009	4.68 (2.13–10.25)	<0.001	4.07 (2.07–8.02)	<0.001
**Presence of a hand-washing station with soap/detergent/ash at/near the latrine (observed) (*n* = 279)**							
Yes	136 (48.75)	Ref.		Ref.		Ref.	
No	143 (51.25)	2.51 (1.06–5.95)	0.036	4.28 (1.80–10.22)	0.001	3.10 (1.52–6.29)	0.002
**Self-reported materials used to wash hands after defecation (*n* = 667)**							
Soap or detergent	278 (41.68)	Ref.		Ref.		Ref.	
Soil/mud/ash/water only	389 (58.32)	1.19 (0.76–1.87)	0.427	1.13 (0.76–1.67)	0.528	1.09 (0.76–1.55)	0.480
**Primary source of bathing water (*n* = 667)**							
Piped water	98 (14.69)	Ref.		Ref.		Ref.	
Hand pump/protected dug well	285 (42.73)	1.41 (0.59–3.44)	0.433	1.12 (0.58–2.15)	0.722	1.16 (0.64–2.08)	0.619
River/canal/pond/unprotected dug well	284 (42.58)	3.57 (1.57–8.11)	0.002	2.00 (1.06–3.74)	0.030	2.23 (1.26–3.94)	0.006

Unadjusted bivariate associations of each of these sanitation factors with APOs are presented in Table 2. Compared to latrine access, open defecation was associated with higher odds of APO (OR: 2.53; 95% CI: 1.72–3.71), preterm birth (OR: 2.36; 95% CI: 1.54–3.62), and low birth weight (OR: 2.00; 95% CI: 1.24–3.23). Risk of APO (OR: 3.92; 95% CI: 1.80–8.52) was also considerably higher for women who used a latrine only occasionally. Water not being available at the latrine was also associated with an increased odds of APO (OR: 4.07; 95% CI: 2.07–8.02). Women who reported bathing or body washing with an open source of water such as a pond, river, or canal also had significantly higher odds of APO (Table 2).

Table 3 shows the results of the multivariable model adjusted for covariates that were selected using *a priori* criteria, as related to latrine access and APOs. The model estimate in the multivariable analysis for the association of open defecation with APO (AOR: 2.38; 95% CI: 1.49–3.80) was minimally attenuated (Table 3). We also observed that higher wealth index was not associated with a reduction in the odds of APOs (AOR: 0.97; 95% CI: 0.80–1.18); however, higher education was found to be associated with a reduction in the odds of APO (AOR: 0.68; 95% CI: 0.59–0.79). Higher haemoglobin in the first trimester was found to be significantly associated with lower odds of APOs (AOR: 0.50; 95% CI: 0.31–0.81) (Table 3). We further investigated the association between latrine use and APOs among participants with latrine access (Table 4). Our results specifically demonstrate that latrine access alone is not associated with a reduction in the burden of APOs; however, latrine use is. Our model estimated 7-fold higher odds of APOs among pregnant women who had access to a latrine but used it only rarely (AOR: 7.10; 95% CI: 2.18–23.11) compared to women who used a latrine often/daily. The association of poor sanitation practices with APOs was independent of poverty in our study settings.

**Table 3:** Multivariable adjusted models for the association between sanitation characteristics and adverse pregnancy outcomes (*n* = 667).

Characteristic	*n* (Percent)	Low Birth Weight (*n* = 95)		Premature Birth (*n* = 130)		APO (*n* = 172)	
		AOR (95% CI)	*p*-Value	AOR (95% CI)	*p*-Value	AOR (95% CI)	*p*-Value
**Access to latrine**							
Latrine in house/neighbour’s house	279 (41.83)	Ref.		Ref.		Ref.	
No latrine—go to open field/bush	388 (58.17)	1.61 (0.94–2.73)	0.078	2.22 (1.29–3.79)	0.004	2.53 (1.72–3.71)	<0.001
**Materials used to wash hands after defecation**							
Soap or detergent	278 (41.68)	Ref.		Ref.		Ref.	
Soil/mud/ash/water only	389 (58.32)	1.39 (0.85–2.27)	0.182	1.29 (0.79–2.11)	0.293	1.22 (0.79–1.88)	0.353
**Primary source of bathing water**							
Piped water	98 (14.69)	Ref.		Ref.		Ref.	
Hand pump/protected dug well	285 (42.73)	1.34 (0.53–3.38)	0.528	0.98 (0.43–2.22)	0.970	0.94 (0.46–1.91)	0.877
River/canal/pond/unprotected dug well	284 (42.58)	2.88 (1.15–7.18)	0.023	1.11 (0.48–2.53)	0.799	1.27 (0.62–2.60)	0.509
**Place of residence**							
Coastal (rural)	203 (30.43)	Ref.		Ref.		Ref.	
Inland (tribal)	464 (69.57)	0.70 (0.23–2.04)	0.515	1.40 (0.52–3.76)	0.503	0.99 (0.41–2.37)	0.998
**Other socio-economic and anthropometric factors**							
Wealth index		1.01 (0.81–1.26)	0.868	0.96 (0.77–1.20)	0.761	0.97 (0.80–1.18)	0.814
Education		0.67 (0.57–0.80)	<0.001	0.76 (0.64–0.89)	0.001	0.68 (0.59–0.79)	<0.001
Maternal age		1.82 (1.33–2.50)	<0.001	1.71 (1.24–2.38)	0.001	1.57 (1.17–2.10)	0.002
Parity		0.64 (0.51–0.80)	<0.001	0.68 (0.54–0.85)	0.001	0.67 (0.55–0.82)	<0.001
BMI		0.61 (0.39–0.98)	0.041	0.65 (0.41–1.05)	0.081	0.57 (0.38–0.87)	0.010
Haemoglobin		0.48 (0.27–0.85)	0.013	0.61 (0.35–1.05)	0.076	0.50 (0.31–0.81)	0.005
ANC visits completed at birth		0.74 (0.47–1.16)	0.202	0.11 (0.07–0.17)	<0.001	0.17 (0.12–0.26)	<0.001

Models adjusted for the factors in the table, religion (Hindu, Muslim, and Christian), and caste (general, other backward class, schedule caste, and schedule tribe). Parameterization in the multivariate model included place of residence (coastal and inland) maternal age (continuous, years), parity (0, 1, 2, and ≥3), BMI (underweight, normal, overweight), haemoglobin (continuous), number of ANC visits completed at birth (continuous), wealth index (continuous), and education (continuous).

**Table 4:** Association between sanitation use and adverse pregnancy outcomes among participants with latrine access (*n* = 279).

Characteristic	*n* (Percent)	Low Birth Weight (*n* = 95)		Preterm Birth (*n* = 130)		APO (*n* = 172)	
		AOR (95% CI)	*p*-Value	AOR (95% CI)	*p*-Value	AOR (95% CI)	*p*-Value
**Latrine use**							
Often/daily	128 (45.88)	Ref.		Ref.		Ref.	
Few times a week	89 (31.90)	4.19 (0.81–21.59)	0.086	5.51 (1.14–26.55)	0.033	4.93 (1.38–17.54)	0.014
Rarely/as needed	62 (22.22)	7.71 (1.81–32.73)	0.006	9.60 (2.34–39.31)	0.002	7.10 (2.18–23.11)	0.001
**Latrine type**							
Ventilated improved pit latrine	46 (16.49)	Ref.		Ref.		Ref.	
Flush/pour flush to septic tank	31 (11.11)	1.00 (0.10–9.40)	0.999	0.46 (0.04–5.17)	0.537	0.36 (0.05–2.54)	0.308
Simple pit latrine/composting/dry latrine	202 (72.40)	1.36 (0.20–8.98)	0.747	1.02 (0.19–5.45)	0.979	0.80 (0.18–3.43)	0.769
**Water available at latrine**							
Yes	172 (61.65)	Ref.		Ref.		Ref.	
No	107 (38.35)	1.37 (0.33–5.53)	0.658	1.65 (0.42–6.39)	0.464	1.38 (0.43–4.43)	0.587
**Visible faecal contamination at latrine**							
No	219 (78.49)	Ref.		Ref.		Ref.	
Yes	60 (21.51)	2.90 (0.70–11.96)	0.140	1.50 (0.42–5.36)	0.532	3.20 (0.99–10.29)	0.051
**Presence of a hand-washing station with soap/detergent/ash at/near the latrine**							
Yes	143 (51.25)	Ref.		Ref.		Ref.	
No	136 (48.75)	1.88 (0.45–7.77)	0.381	3.47 (0.82–14.70)	0.090	2.11 (0.63–7.03)	0.223

Models adjusted for the factors in the table, place of residence (coastal and inland) maternal age (continuous, years), parity (0, 1, 2, and ≥3), BMI (underweight, normal, overweight), haemoglobin (continuous), number of ANC visits completed at birth (continuous), wealth index (continuous), education (continuous), religion (Hindu, Muslim, and Christian), and caste (general, other backward class, schedule caste, and schedule tribe).

## Discussion

In this prospective study, we examined the relationship between maternal sanitation behaviour and APOs. After adjusting for socio-demographic, anthropometric, and other sanitation-related behaviours, we observed that women who reported poor sanitation practices in the early phase of pregnancy (10–12 wk of gestation) were more likely to experience an APO, independent of the established confounding factors of poverty and caste. To our knowledge, this is the first community-based prospective study to demonstrate that practicing open defecation is associated with a higher risk of APOs. Using a large existing population-based cohort, all pregnancies could be enrolled and followed longitudinally from the first trimester to pregnancy. The diverse nature of our study sites also allowed us to enrol a representative rural Indian sample including individuals of different castes, class, religion, and socio-economic status, with a range of sanitation-related practices.

Population-based data on the prevalence of APOs as reported here are uncommon in India. Among our cohort, we found that 19.5% of births were preterm and 14.2% of babies had low birth weight during the study period. Studies from south India (Tamil Nadu) have reported a prevalence of low birth weight and preterm birth of 17.0% and 12.3%, respectively, although the prevalence of low birth weight has also been estimated to be as high as 30%–40% in India [57]. The rate of preterm delivery (<37 wk) in neighbouring Bangladesh [24] was 22.3% in a setting similar to India. In sub-Saharan Africa, the prevalence of preterm birth and low birth weight in an urban setting was 19.9% and 10.2%, respectively [56].

Given the paucity of research linking open defecation to APOs, it is difficult to assess our findings in the context of other study findings. A hospital-based study in sub-Saharan Africa found a higher risk of preterm birth, but not low birth weight, among babies born to mothers using shared sanitation facilities (OR: 1.26; 95% CI: 1.07–1.48) [56].

Our study reports a protective role of maternal haemoglobin (Hb ≥ 110 g/l) in regards to the risk of APOs among the study populations which is in line with other findings. A recent systematic review and meta-analysis showed a significantly higher risk of low birth weight (OR: 1.29; 95% CI: 1.09–1.53) and preterm birth (OR: 1.21; 95% CI: 1.13–1.30) with anaemia in the first or second trimester [47]. The same study also estimated that each 1-g/l increase in mean haemoglobin corresponded to birth weight being increased by 14.0 g (95% CI: 6.8–21.8). Another study in India revealed 30% of women to be anaemic (Hb < 110 g/dl), and maternal anaemia predicted a 2.4-fold greater risk of preterm delivery (*p* < 0.01) and an increased risk of low birth weight (*p* = 0.05) [16]. We observed that higher haemoglobin was associated with low odds (AOR: 0.50; 95% CI: 0.31–0.81) of APOs among women in the multivariable model.

Another important finding of our study was that education remained a key determinant of APOs in the multivariable model. We observed higher education to be associated with lower odds (AOR: 0.68; 95% CI: 0.59–0.79) of APOs among women in the multivariable model. This finding is consistent with numerous research findings of higher education being predictive of lower likelihood of APOs [31,61]. A Canadian study showed that not having a high school diploma was associated with low birth weight (OR: 3.20; 95% CI: 2.61–3.91). The inverse association of education with APOs could be attributed to many socio-behavioural factors related to the overall impact of higher levels of education. Education can improve knowledge about safe hygiene practices and assimilation of health-related information and good hygiene behaviour [62,63]. Similarly, an educated person may be more likely to appreciate the positive effect of sanitation and hygiene practices, resulting in sustained healthy behaviour [64].

We adjusted our estimates to account for socio-economic factors by constructing a household wealth index. Poverty is highly correlated with a lack of sanitation access, and both factors have been linked to increased risks regarding maternal health [31,32], stunting [41], and stress. While it is intuitive to expect that individuals with low economic status are more likely to experience APOs because of many concomitant negative determinants of pregnancy, our results demonstrate that open defecation poses significant health risks that are not explained by poverty.

In our study, although we adjusted our estimates for *a priori* covariates and considered several biological plausibilities, we did not adjust for many potential risk factors for APOs such as maternal smoking, alcohol use, history of sexually transmitted diseases, history of antenatal complications, history of abortions, etc. Whereas each of these factors has been associated with an increased risk of APOs, we are unaware of any data suggesting that these variables are associated with defecation practice. Hence, we considered these variables unlikely to be confounders in our analysis [60]. Several questions remained unanswered in this study, and we have not been able to address the biological or behavioural basis of these findings. One mechanism may be related to the adverse outcomes of restricting food and water intake to cope with sanitation challenges. It has been shown that women, when confronted with poor sanitation choices, may choose to limit their intake of food and water to avoid the need to use the toilet. Another potential mechanism may be related to community-level or household-level coverage and use of sanitation and the resulting increase in disease prevalence associated with environmental contamination in drinking water or in food. The pathogenesis of each of the adverse birth outcomes is unique and potentially independent, and we have not identified the specific exposure pathways (such as incidence of bacterial vaginosis) that may play a role in our observed outcomes. A third potential mechanism may be related to the lack of sanitation and psycho-social stress. Women throughout their life course may experience tangible threats to physical health as a result of sanitation insecurity, a mechanism currently being explored as part of this study.

This study indicates that, in the context of maternal and child health promotion research, sanitation is an important dimension of women’s health and distinct from poverty and caste. Additional research is warranted that addresses the underlying mechanisms of sanitation-related APOs.

## Supporting Information

S1 Data Dataset and codebook. (ZIP)

S1 Fig Location map of the study settings. (TIF)

S1 Table Distribution of wealth index scores. (DOCX)

S1 Text Prospective statistical analysis plan. (DOC)

S2 Text STROBE statement. (DOC)

## Abbreviations:

ANC: antenatal care
AOR: adjusted odds ratio
APO: adverse pregnancy outcome
BMI: body mass index
CHV: community health volunteer
Hb: haemoglobin
JMP: WHO/UNICEF Joint Monitoring Programme for Water Supply and Sanitation
OR: odds ratio
WASH: water, sanitation, and hygiene
WHO: World Health Organization
