# Supplementary figures and images for: Risk of Adverse Pregnancy Outcomes among Women Practicing Poor Sanitation in Rural India: A Population-Based Prospective Cohort Study
Source: PLoS Med. 2015 Jul 7;12(7):e1001851. doi: 10.1371/journal.pmed.1001851 (PMC4511257; doi:10.1371/journal.pmed.1001851)

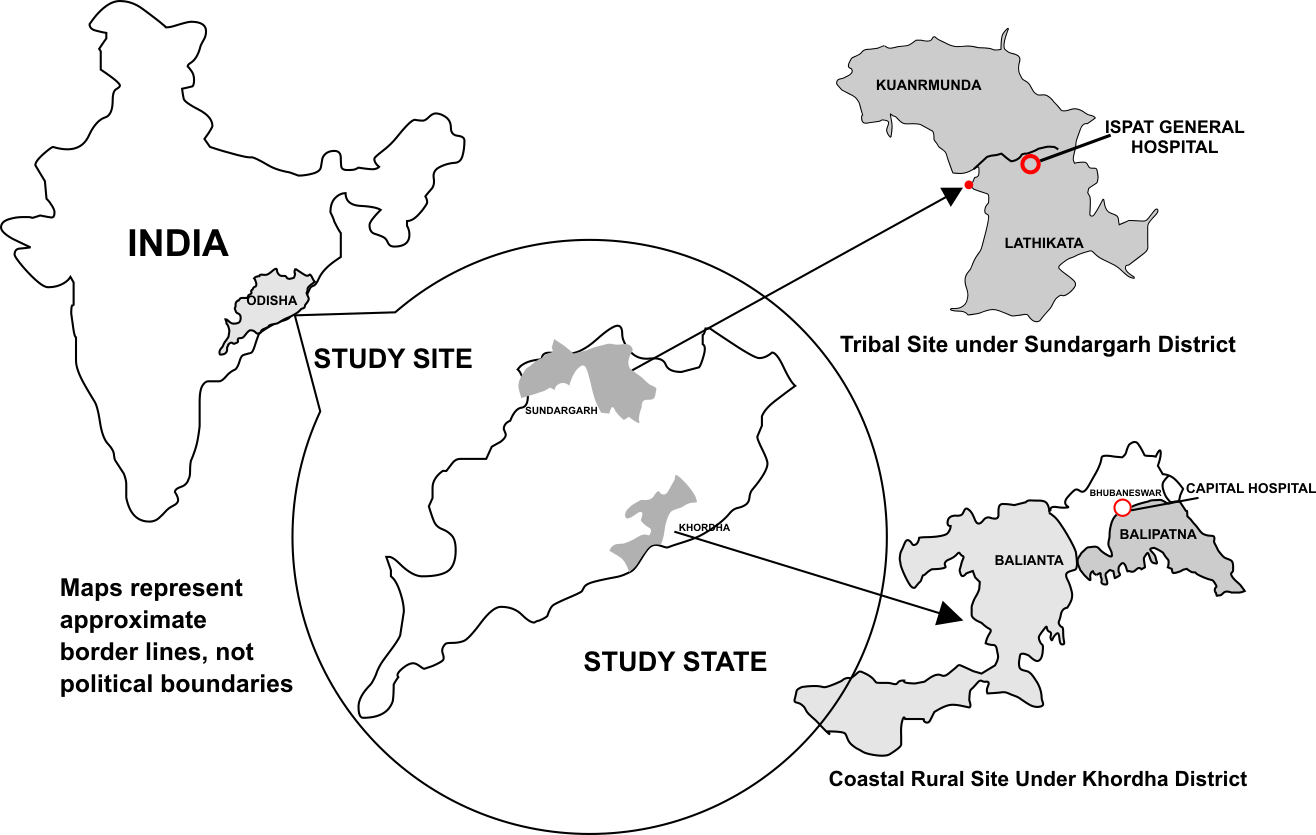

Supplement: S1 Fig — (TIF) [file pmed.1001851.s002.tif]
