# Supplementary material for: Risk of Adverse Pregnancy Outcomes among Women Practicing Poor Sanitation in Rural India: A Population-Based Prospective Cohort Study
Source: PLoS Med. 2015 Jul 7;12(7):e1001851. doi: 10.1371/journal.pmed.1001851 (PMC4511257; doi:10.1371/journal.pmed.1001851)
Supplement: S1 Table — (DOCX) [file pmed.1001851.s003.docx]

**Table S1. Distribution of wealth index scores.**

| Wealth quartiles/categories | Mean | SD | Minimum | Maximum |
| --- | --- | --- | --- | --- |
| Quartile 1 | -3.443257 | 0.452751 | -4.422642 | -2.830487 |
| Quartile 2 | -0.3667073 | 1.376385 | -2.813859 | 1.13693 |
| Quartile 3 | 1.644051 | 0.2924501 | 1.142057 | 2.080589 |
| Quartile 4 | 2.219495 | 0.0825456 | 2.091028 | 2.458026 |
