# Supplementary material for: Risk of Adverse Pregnancy Outcomes among Women Practicing Poor Sanitation in Rural India: A Population-Based Prospective Cohort Study
Source: PLoS Med. 2015 Jul 7;12(7):e1001851. doi: 10.1371/journal.pmed.1001851 (PMC4511257; doi:10.1371/journal.pmed.1001851)
Supplement: S1 Text — (DOC) [file pmed.1001851.s004.doc]

**S1- Padhi BK et al. Statistical Analysis Plans for “Risk of adverse pregnancy outcomes among women practicing poor sanitation: a population-based prospective study”**

**STUDY DESIGN**

This study is a prospective independent cohort, observational study examining the relationship between poor sanitation practices and adverse pregnancy outcomes.

**Objective (s)**

The primary aim of the study is to assess the risk of APO associated with poor sanitation access and practices using a population-based pregnant cohort, with specific aims:

1) To quantify the prevalence of open defecation practices among pregnant women at their first trimester.

2) To investigate the relationship between poor sanitation practices and adverse pregnancy outcomes.

**Study Settings**

Coastal rural (Balianta & Balipatana Blocks of Khurda District) and Mainland tribal (Kuarmunda and Lathikata Blocks of Sundargarh District) in Odisha, India.

**Sample Size and Power of the Study**

Previous research conducted at AIPH suggests that birth of a low birth weight infant occurs in approximately 20% of all pregnant women, of which 12-14% are preterm. We estimate that a sample size of 582 women should give us 80% power to detect a 1.5 fold increase in risk of APO if the mother practices open defecation during her first trimester of pregnancy, at a significance level of 95% (using a two tailed test). Estimating an anticipated 15% dropout, our final sample size is planned to be 670. The sample size was calculated using the following formula (Fleiss, 1981)

INCLUDEPICTURE "http://www.statsdirect.com/help/content/image/stat0072_wmf.gif" \* MERGEFORMATINET
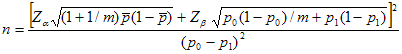


Where

INCLUDEPICTURE "http://www.statsdirect.com/help/content/image/stat0073_wmf.gif" \* MERGEFORMATINET
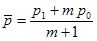


Z-alpha= 1.96 at the for alpha of 0.05 i.e. 95% confidence level

Z-beta=0.84 for beta of 0.2 i.e. power of 80%

*P_0_* is the probability of events in the unexposed (which is approximately 20% APO in our study)

*P_1_* is the probability of events in exposed (which is estimated to be 30% i.e. 1.5 times in our study)

m= ratio of exposed to unexposed which is approximately 1:1 in our study as the unfavourable sanitation practices (mainly open defecation is believed to be 50%).

**Study Outcome Measures**

**A) Primary:** The primary outcome of interest will be the incidence of an adverse pregnancy outcome (APO), defined as an event of either preterm birth (PTB), low birth weight (LBW), or both, spontaneous abortion, and still birth. We will use World Health Organization (WHO) definitions for all primary outcome measures. Preterm birth will be defined as birth of a live baby before 37 completed weeks of gestation or fewer than 259 days since the first day of the woman’s last menstrual period. Neonates weighing < 2500 grams at birth will be considered to be of LBW. Spontaneous abortion will be defined as “fetal loss” before 22 weeks of gestation with gestational age estimated from the participants’ self-reported last menstrual period. Stillbirth will be defined as fetal death after 22 weeks gestation with no sign of life (cord pulsation or muscle movement). Infant demographics such as gestational age, birth weight, and gender of the child should be abstracted from medical charts at delivery by the CHV. The birth data should be recorded into a customized mother-baby tracking cards provided to all pregnant women for future follow-up purposes.

Infant demographics such as gestational age, birth weight, and gender of the child will be abstracted from the birth records by the CHV at the delivery and should be entered individual mother-baby cards. For interpretation purposes we will classify the outcomes into three categories (APO, PTB and LBW). All reported adverse pregnancy outcomes (PTB, LBW, stillbirth and abortions) will be grouped into one category and will be referred as “adverse pregnancy outcomes (APO)”; PTB and LBW may be interpreted separately.

**B) Secondary:** We anticipate studying the following secondary outcomes using the same cohort:

1) Incidence of change of sanitation behaviour

2) Incidence of UTI/BV symptoms

3) Prevalence of anaemia and BMI status among the pregnant cohort

4) Incidence of morbidities such as malaria, gastrointestinal illness etc.

5) Key social, cultural, and economic considerations that serve as motivators and barriers among pregnant cohort to adopt improved sanitation and hygiene practices

**Study Exposure Measures**

**A) Primary exposure assessment:** A household survey with observation (checklist) will be conducted by trained by CHVs addressing specific questions on defecation and hygiene practices and conditions during recruitment and subsequent visits. A similar question will also be asked to record the sanitation practices before pregnancy. To document the number of women using a latrine and variability in their practice, participants will be asked to identify the primary and secondary locations were they had defecated in the last month, and whether there were instances in the past month where they chose not to use the primary defecation site. Visual observations of defecation sites will be performed to confirm the interview response, to inspect for the presence of a functional water source or water storage container at the latrine, and where a latrine was used to document the type of latrine design and inspect for the presence of visible fecal contamination on latrine floors, walls, and stances. Other information such as hand washing practice after defecation, source of bathing water will also be collected.

**B) Collection of Covariates/confounders:** A face to face interview along with personal measurements will be conducted using a structured questionnaire by a CHV to collect maternal age, socio-demographic information, mother's anthropometric characteristics such as height and weight. Ownership of BPL card (a proxy measure for poverty), household characteristics and assets will also be recorded. Maternal hemoglobin at recruitment will be collected from Mother & Child Tracking System card. For women, where this information unavailable, a study supervisor will conduct the haemoglobin estimation from finger prick blood samples using a portable haemoglobin analyzer (HemoCue® Hb 301).

**STATISTICAL ANALYSIS PLAN**

The study will employ an independent prospective cohort, where a random sample (N=670 pregnant women of 10-12 weeks of gestation) will be selected from the population census. Descriptive statistics (frequency, means, and standard deviations) will be calculated for all demographic, anthropometric, clinical outcomes and baseline sanitation behavior variables. The outcome that our analysis will principally work with includes Adverse Pregnancy Outcome (APO) which includes preterm birth (PTB) and low birth weight (LBW), using definitions described previously. The relationship between the main explanatory variable in our study that includes sanitation access and practice, and the principal outcomes (APO, PTB and LBW), all of which are binary will initially be examined using contingency tables and differences in distribution of outcomes across exposure groups tested using Chi-square test. The risk of APOs contingent upon sanitation access and practice will be estimated using appropriate regression analysis. The models will be adjusted for co-variates which are considered *a priori* to be confounders of this relationship, which includes maternal age; BMI, maternal haemoglobin as indicator of maternal nutritional status; and household poverty and educational attainment of the mother as indicators of maternal socio-economic status. Alpha will be set at 0.05 for all tests of hypotheses.

**Multiple comparisons and multiplicity**

Results from all analyses will be assessed against an alpha of 0.05. There will be no adjustments for multiplicity as there are pre-defined outcomes and objectives, and the influence of individual results on the overall interpretation of the study will reflect their importance.

**Covariate adjustment**: The primary statistical analyses for the outcome variables “APO” measured at the follow-up visit will be adjusted by performing analyses adjusted for other baseline characteristics suspected *a priori* to be associated with the outcome. Any group differences in potentially influential baseline characteristics will be included in the interpretation and discussion of results.

**Incomplete follow-up:** For each subject, participation in the study will end at birth/pregnancy outcomes. If a subject migrates or withdraws, data collected until that point in time will be used.

**Missing outcome data:** All precautions should be made for no missing values on baseline variables that are planned for use as covariates. For the primary outcome and secondary outcomes, subject who do not have a measurement taken at the baseline or subsequent follow-up visit will be restricted from the final analysis.

**Outliers:** Outliers will be identified by examining residual plots. Cases that visually “stand out” will be assessed for a possible influence on results. A comparison of results from analyses with and without the outlier(s) may be performed. Where there are discrepant results from the two analyses, this will be reported and discussed in the manuscript(s).

**Data management and analysis software**

Database will be created using Epi-Info custom-designed database management interface. Most data manipulation, tables, figures, listings and analyses will be documented in Stata programs and performed using Stata® Software version 13.

**Data confidentiality**

All the collected data from the participants of the study will be in line with the Health Insurance Portability and Accountability Act of 1996 (HIPAA). HIPAA establishes security and privacy standards for the use and disclosure of “protected health information” (PHI) of study participants. Only de-identified data will be used for further analysis.

**Quality Control and Quality Assurance**

Quality of data collected by CHVs will be ensured through direct supervision and random checking by respective field supervisors, and further checking of a subset by the project manager. Supervisory visits and standardization exercise sessions will be organized routinely to ensure quality of data collection. Every reported outcome of interest will be confirmed by a repeat visit to the household by a supervisory staff. A sample (10%) of collected forms will be cross-checked by the supervisory team. Data will be double entered to a custom-designed database management interface using Epi-Info. Some data points will be randomly selected for comparison between the paper report and the electronic database. Field verifications will be conducted to resolve identified inconsistencies and incompleteness as and when required.
