# Supplementary material for: Risk of Adverse Pregnancy Outcomes among Women Practicing Poor Sanitation in Rural India: A Population-Based Prospective Cohort Study
Source: PLoS Med. 2015 Jul 7;12(7):e1001851. doi: 10.1371/journal.pmed.1001851 (PMC4511257; doi:10.1371/journal.pmed.1001851)
Supplement: S2 Text — (DOC) [file pmed.1001851.s005.doc]

**S2 – Padhi BK et al. STROBE Statement:** Risk of adverse pregnancy outcomes among women practicing poor sanitation: a population-based prospective study

|  | Item No | Recommendation |
| --- | --- | --- |
| **Title and abstract** | 1 | (*a*) Indicate the study’s design with a commonly used term in the title or the abstract  **Abstract, Methods and Findings: A prospective cohort of pregnant women (N= 670) in their first trimester … association was independent of poverty, caste, and religion** |
|  |  | (*b*) Provide in the abstract an informative and balanced summary of what was done and what was found  **A trained community health volunteer (CHV) visited the homes to conduct follow-up visits to record the pregnancy outcomes. Adjusted odds ratios (OR) and 95% confidence intervals (95% CI) for adverse pregnancy outcomes were estimated by logistic regression models. Of the 667 women who completed the follow-ups, 58.2% practiced open defecation and 25.7% experienced adverse pregnancy outcomes, including 130 (19.4%) preterm birth, 95 (14.2%) low birth weight, 11 (1.7%) spontaneous abortions and 6 (0.9%) stillbirths. After adjustment for potentially confounders such as maternal socio-demographics and clinical factors, open defecation was significantly associated with increased risk of adverse pregnancy outcomes (odds ratio [OR]: 2.38; 95% CI: 1.49-3.80) preterm birth (OR: 2.22; 95% CI: 1.29-3.79) and low birth weight (OR: 1.61; 95% CI: 0.94-2.73). The association was independent of poverty, caste, and religion.** |
| Introduction | | |
| Background/rationale | 2 | Explain the scientific background and rationale for the investigation being reported  **Introduction: The burden of adverse pregnancy outcomes (APO), which includes both preterm births low birth weights … (through) … are extremely limited.** |
| Objectives | 3 | State specific objectives, including any prespecified hypotheses  **Last paragraph of introduction: “… … with specific aims to quantify the prevalence of open defecation practices among pregnant women and its contribution to adverse pregnancy outcomes”** |
| Methods | | |
| Study design | 4 | Present key elements of study design early in the paper  **See answer to “objectives” item 3 above, expanded in Methods with a section “study design”.** |
| Setting | 5 | Describe the setting, locations, and relevant dates, including periods of recruitment, exposure, follow-up, and data collection  **Methods: The state of Odisha, home to 41.9 million people, has one of the highest infant (57 per 1,000 live births) and maternal mortality (257 per 100,000) in India …… (through)….. Each study site had one CHV responsible for following 4-5 pregnancies, supervised by an area coordinator (1 per~10 CHVs), reporting to a program manager.** |
| Participants | 6 | (*a*) *Cohort study*—Give the eligibility criteria, and the sources and methods of selection of participants. Describe methods of follow-up  **Methods: Study design- We conducted a population-based prospective cohort study ……** **A schematic diagram of the study design is shown in Figure 1. Baseline and three follow-ups were conducted during the course of pregnancy (one in second and two in third trimester), followed by one at birth.**  *Case-control study*—Give the eligibility criteria, and the sources and methods of case ascertainment and control selection. Give the rationale for the choice of cases and controls  *Cross-sectional study*—Give the eligibility criteria, and the sources and methods of selection of participants |
|  |  | (*b*) *Cohort study*—For matched studies, give matching criteria and number of exposed and unexposed  *Case-control study*—For matched studies, give matching criteria and the number of controls per case |
| Variables | 7 | Clearly define all outcomes, exposures, predictors, potential confounders, and effect modifiers. Give diagnostic criteria, if applicable  **In methods section there are specific subheadings of exposure, outcome covariates and confounders** |
| Data sources/ measurement | 8* | For each variable of interest, give sources of data and details of methods of assessment (measurement). Describe comparability of assessment methods if there is more than one group  **In Method section: The survey instruments (questionnaires, and observation checklists) were developed……… (through)…..** **a study supervisor conducted anthropometric recordings and haemoglobin estimation from finger prick blood samples using a portable haemoglobin analyzer (HemoCue® Hb 301).** |
| Bias | 9 | Describe any efforts to address potential sources of bias  **Potential major biases such as attrition and missing variables were addressed during the design stage and every effort was made during data collection to collect full information. These have been addressed in the third paragraph of method section and design section.** |
| Study size | 10 | Explain how the study size was arrived at  **In method section: Considering 20.0% prevalence of APO, the sample size was calculated to be 582 to detect a relative risk of 1.5 with 95% confidence at 80% power. All pregnant women satisfying the eligibility criteria in the study population were recruited in the study. Estimating an anticipated 15.0% dropout, our final sample size was 670. There were 708 eligible pregnant women in the two study settings (coastal and mainland). A random number generator selected the 670 geocoded households/women who were enrolled through a household visit by the CHV.** |
| Quantitative variables | 11 | Explain how quantitative variables were handled in the analyses. If applicable, describe which groupings were chosen and why  **We used World Health Organization definitions for all outcome measures. Preterm birth was defined as birth of a live baby before 37 completed weeks of gestation or fewer than 259 days since the first day of the woman’s last menstrual period. Neonates weighing < 2500 grams at birth were considered to be of LBW. Spontaneous abortion was defined as “fetal loss” before 22 weeks of gestation with gestational age estimated from the participants’ self-reported last menstrual period. Stillbirth was defined as fetal death after 22 weeks gestation with no sign of life (cord pulsation or muscle movement).** |
| Statistical methods | 12 | (*a*) Describe all statistical methods, including those used to control for confounding  **In methods, a separate section on “Statistical analysis”: We conducted cross tabulation to explore frequencies…(through)….** **The data were analysed using STATA (version 13) statistical package.** |
|  |  | (*b*) Describe any methods used to examine subgroups and interactions  **No interaction terms has been tested** |
|  |  | (*c*) Explain how missing data were addressed  **There were no missing values in the study.** |
|  |  | (*d*) *Cohort study*—If applicable, explain how loss to follow-up was addressed  *Case-control study*—If applicable, explain how matching of cases and controls was addressed  *Cross-sectional study*—If applicable, describe analytical methods taking account of sampling strategy  **Only three cases were lost to follow-up and excluded from the analysis. At the design stage we have included a higher sample size to address the issue.** |
|  |  | (*e*) Describe any sensitivity analyses  **NA** |

| Results | | |
| --- | --- | --- |
| Participants | 13* | (a) Report numbers of individuals at each stage of study—eg numbers potentially eligible, examined for eligibility, confirmed eligible, included in the study, completing follow-up, and analysed  **See Study size, item 10 above. Of the 670 women recruited into the study, 667 completed the study, of which 172 (28.2%) experienced adverse pregnancy outcomes (Figure 1).** |
|  |  | (b) Give reasons for non-participation at each stage: **NA** |
|  |  | (c) Consider use of a flow diagram **(Figure 1).** |
| Descriptive data | 14* | (a) Give characteristics of study participants (eg demographic, clinical, social) and information on exposures and potential confounders  **Table 1 and in result section “Among these women, 130 (19.4%) were preterm birth, 95 (14.2%) low birth weight, 11 (1.6%) spontaneous abortions and 6 (0.9%) still births. Detailed socio-demographic, anthropometric and clinical characteristics of study participants and their associations with preterm birth and low birth weight are shown in Table 1.”** |
|  |  | (b) Indicate number of participants with missing data for each variable of interest  **NA** |
|  |  | (c) *Cohort study*—Summarise follow-up time (eg, average and total amount)  **All pregnant women were followed from their first trimester (12 weeks of gestation) till birth.** |
| Outcome data | 15* | *Cohort study*—Report numbers of outcome events or summary measures over time  **Results, first paragraph and Figure 1, item 14 (a) above.** |
|  |  | *Case-control study—*Report numbers in each exposure category, or summary measures of exposure |
|  |  | *Cross-sectional study—*Report numbers of outcome events or summary measures |
| Main results | 16 | (*a*) Give unadjusted estimates and, if applicable, confounder-adjusted estimates and their precision (eg, 95% confidence interval). Make clear which confounders were adjusted for and why they were included  **See Table 2 for unadjusted estimates and confounder-adjusted estimates in Table 3 and Table 4. Analyses were adjusted for maternal age, BMI, hemoglobin, education and poverty using *a priori* hypothesis as well as considering the biological plausibility.** |
|  |  | (*b*) Report category boundaries when continuous variables were categorized **NA** |
|  |  | (*c*) If relevant, consider translating estimates of relative risk into absolute risk for a meaningful time period **NA** |
| Other analyses | 17 | Report other analyses done—eg analyses of subgroups and interactions, and sensitivity analyses  **No sub-group/interactions were tested in the full model.** |
| Discussion | | |
| Key results | 18 | Summarise key results with reference to study objectives  **Discussion, beginning: In this prospective study, we examined the relationship between maternal sanitation behaviour and adverse pregnancy outcomes. After adjusting for socio-demographic, anthropometric and other hygiene-related behavior, we found that women who reported open defecation practice in the early phase of pregnancy (10-12 weeks of gestation) were more likely to experience adverse birth outcomes, independent of established confounding factors.** |
| Limitations | 19 | Discuss limitations of the study, taking into account sources of potential bias or imprecision. Discuss both direction and magnitude of any potential bias  **Discussion, section paragraph#8: “Even though we have accounted for several key confounding ….(through)….** **bacterial vaginosis (BV) and sexual transmitted infections (STIs).** |
| Interpretation | 20 | Give a cautious overall interpretation of results considering objectives, limitations, multiplicity of analyses, results from similar studies, and other relevant evidence  **Discussion, section last paragraph: “This study indicates that in the context of maternal and child health prevention research, sanitation is an important dimension of women’s health and distinct from social class and caste. Additional research into the role of these mechanisms is warranted.”**  **A subheading “interpretation” is also presented at the very beginning of the article “Abstract”.** |
| Generalisability | 21 | Discuss the generalisability (external validity) of the study results  **Discussion section, first paragraph: “Using an existing large population-based cohort, all pregnancies could be enrolled and followed longitudinally from first trimester to pregnancy. Due to the diverse nature of our study sites, this study also allowed us to enroll a representative rural Indian sample including different castes, class, religion, and socio-economic status, with a range of sanitation-related practices.”** |
| Other information | | |
| Funding | 22 | Give the source of funding and the role of the funders for the present study and, if applicable, for the original study on which the present article is based  **Funding from the study was obtained from the Department for International Development-backed SHARE Research Consortium and Water Supply and Sanitation Collaborative Council (WSSCC). The funders had no role in study design, data collection and analysis, decision to publish, or preparation of the manuscript.** |

*Give information separately for cases and controls in case-control studies and, if applicable, for exposed and unexposed groups in cohort and cross-sectional studies.

**Note:** An Explanation and Elaboration article discusses each checklist item and gives methodological background and published examples of transparent reporting. The STROBE checklist is best used in conjunction with this article (freely available on the Web sites of PLoS Medicine at http://www.plosmedicine.org/, Annals of Internal Medicine at http://www.annals.org/, and Epidemiology at http://www.epidem.com/). Information on the STROBE Initiative is available at [www.strobe-statement.org](http://www.strobe-statement.org/).
